# Media Analysis of News Articles During COVID-19: Renewal, Continuity and Cultural Dimensions of Creative Action

**DOI:** 10.3389/fpsyg.2020.601938

**Published:** 2021-02-16

**Authors:** David Mattson, Katie Mathew, Jen Katz-Buonincontro

**Affiliations:** School of Education, Drexel University, Philadelphia, PA, United States

**Keywords:** creativity, socio-cultural theory, COVID-19, quarantine, media analysis

## Abstract

Worldwide, the COVID-19 pandemic has forced people to adapt quickly, and to reexamine interactions and responsibilities toward communities in creative ways. This paper presents a qualitative media analysis (Altheide and Schneider, 2013) of 50 online news articles (*Los Angeles Times and New York Times*) published between March 17th and August 6th, 2020 using the key-words “creativity” and “COVID-19.” Informed by a definition of creativity as actions that are considered both “new” and “appropriate” (Sternberg and Lubart, 1999), articles describing a “creative action” were kept for analysis. These articles highlight creative responses to the COVID-19 quarantine in various domains including architecture, fashion, and faith. In this paper, we discuss the themes derived during this analysis- “renewal and continuity” and “the multidimensionality of creativity” which elaborate and contextualize a perspective of socio-cultural creativity theory and propose two implications of this study. The first implication posits that creativity was an observable, cultural response to the COVID-19 pandemic. The second implication offers a broader concept of how cultural resources function as dynamic constraints or “affordances” within the Five A’s model of creativity (Glǎveanu, 2013). Discussion of further research through the lens of socio-cultural creativity is discussed.

## Introduction

The saying “necessity is the mother of invention” aptly described the transformation of communities under the disruptions of COVID-19. Due to the pandemic, communities scrambled quickly to come up with creative solutions to the constraints of quarantine lockdowns. Governments, hospitals, businesses, and schools have altered their services or products in “novel” and “appropriate” ways to prevent the spread of the virus (Sternberg and Lubart, 1999). Many prominent creative theories emphasize an individual difference approach to creativity or focus on each singular act as an isolated event. However, in the circumstance of COVID-19, what makes some creative actions remarkable is the cultural determination of their creative value and meaning. Following in the legacy of Clifford Geertz, we utilize a broad definition of culture as “a system of meanings embodied in symbols” such that culture provides individuals with the symbols and meaning necessary for creative action (Geertz, 1973; Yarrow, 2006). This paper presents a study of media analysis of news articles reporting on creativity during COVID-19 to elucidate a socio-cultural perspective of creative actions. We first ground the study in socio-cultural theories of creativity.

### Socio-Cultural Perspective of Creativity

The origins of the socio-cultural perspective of creativity are in the scholarship of Dewey (1934), Vygotsky (1971), Bakhtin (1994), Jaramillo (1996), Sternberg et al. (2019). Vygostky’s theory of internalization and externalization, interpreted by Moran et al. (2003), is particularly relevant. Framing externalization (typically considered the creative output) and the internalization of culture as an ongoing cycle rather than two separate pathways, this process suggests the interdependence of culture and creative action that is foundational to the socio-cultural perspective of creativity (Moran et al., 2003). Dewey’s writing on the artist in constant relationship to his audience also pushed against a fundamentally individualistic view of creativity. “Even when the artist works in solitude all three terms are present; work, artist, and audience… the artist has to become vicariously the receiving audience” (Dewey, 1934, p. 111). This description of an artist creating art with consideration of the audience in his own mind foreshadowed what would become another central tenet of socio-cultural perspectives: creativity never happens apart from the culture that shapes the tools and environment of the creator (Tanggaard, 2013; Glãveanu et al., 2019).

Csikszentmihalyi’s systems model of creativity (1998) further developed the notion of the creative relationship between the individual and society. The systems model identifies individual, domain, and field as the integral pieces or “nodes” of creative production. Key gatekeepers shape what is promoted as “creative” by supporting and accepting creative work or, conversely, rejecting or downplaying creative work (Sawyer et al., 2003). In *Creativity and Development* (2003), Moran and John-Stein draw parallels between Vygotsky and Csikszentmihalyi to highlight social processes in creativity. Csikszentmihalyi’s systems model of creativity (Csikszentmihalyi and Rochbert-Halton, 1981) depends on the idea that individuals rely on social interactions, “(creativity) is constructed through an interaction between producer and audience” (Csikszentmihalyi, 1998, pp. 314). The systems model describes the relationship between the individual and culture in a way that captures their dynamic of interdependence by emphasizing the role of the individual to contribute creative ideas or products while simultaneously requiring culturally situated “domains” and “fields” to accomplish this task (1998). Moran also compares Rhodes concept of “press” from the “Four P’s of Creativity” to Csikszentmihalyi’s idea of “field,” suggesting that both Rhodes and Csikszentmihalyi posit an individual’s cultural interaction as foundational for the existence of creativity. How individuals interact and depend on culture to perform creative action was further expanded through the “Five A’s” theory of creativity (Glaveanu et al., 2013; Glǎveanu, 2015; Sternberg et al., 2019).

### Creative Action

Glǎveanu (2013, 2015) and Glãveanu et al. (2019) provides two critiques of how Rhodes “Four P’s” theory has been used within creativity research. The first criticism is that a focus on these four categories (person, process, product, and press) led to the separation of creativity research into silos of study focused on the individual components rather than their interaction. The second criticism is that these categories “have been studied in ways that decontextualize creativity and do not engage with societal and cultural elements sufficiently” (Glǎveanu, 2013, pp. 71). The “Five A’s” theory (actors, audience, action, artifacts, and affordances) reframes both the categories of creativity and their interaction to account for a socio-cultural perspective of creativity (Glǎveanu, 2013). In particular, the addition of “affordances” rather than “press” demonstrates a shift of perspective. Although the “Five A’s” model may not supplant the “Four P’s” as the dominant model within creativity research, it is the broader lens of socio-cultural perspectives of creativity that shows promise for bringing together researchers who have traditionally viewed creativity from disparate paradigms (Glãveanu et al., 2019).

**FIGURE 1:**
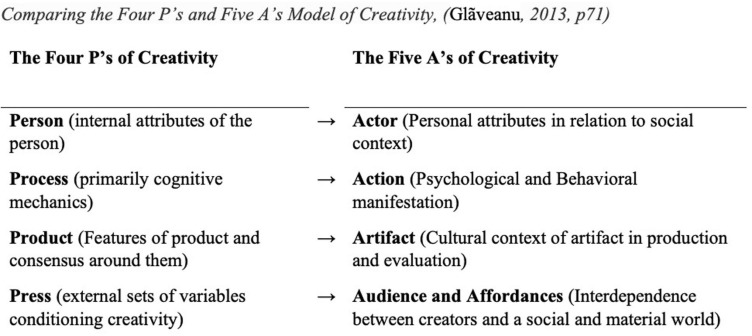
Comparing the Four P’s and Five A’s model of creativity (Glǎveanu, 2013, p. 71). *Creative action during COVID-19 is the focus of the media analysis.

In Figure 1, the term “creative action” is defined as both psychological and behavioral. The term “action” represents both creative ideation and performance; both internalized and externalized (Glǎveanu, 2013). This shift of perspective from “process” to “action” informed the motivation for the present study focusing on creative action as reported in media articles. In order to evaluate creativity as a response to COVID-19, it was necessary to utilize a category of “creativity” possessing theoretical significance while still inclusive of a wide spectrum of creativity often studied as disparate phenomena.

Additionally, the term “affordance” rather than “press” facilitated a more specific vocabulary for describing the interaction of quarantine conditions on creative “action.” Creativity research seldom considers the material world of equal importance to creativity as cognitive processes (Glǎveanu, 2013). The concept of “affordances” raises the material objects and circumstances surrounding individuals as equal with all other aspects of the creative endeavor (Glǎveanu, 2013). COVID-19, and the subsequent quarantine, can be considered an “affordance” that shapes creative “action.” According to the socio-cultural theory of creativity, “culture” is “neither external to the person nor static, but constitutive of the mind and of society by offering the symbolic resources required to perceive, think, remember, imagine, and, ultimately, create” (Glãveanu et al., 2019, pp. 2). If culture is necessary for thought, reason, and imagination, then every artifact derived from these tools is also culturally situated, or a *cultural resource*. These resources are passed down within culture. Tanggaard’s (2013, pp. 02) theory of socio-material creativity posits that everyday creativity draws on these cultural resources “When we are creative, we rarely produce knowledge that is wholly detached from the prior knowledge of ourselves and others.” Creativity is equally present in novelty and in the renewal of what already exists. By incorporating these terms and concepts with a socio-cultural perspective of creativity the researchers possessed the language and theory necessary to interpret and categorize the broadest range of creative actions shaped by the affordances of COVID-19.

### Present Study

In response to the pandemic, our research team witnessed “creative actions”: acts of adaptation, generosity, or personal courage in our personal lives. We wondered how widespread these creative acts were across the United States and decided to explore their prevalence through conducting a media analysis. Media analysis builds on the prior work of qualitative research that focuses on scrutinizing documents that record or characterize human behavior in specific ways (Altheide and Schneider, 2013). Media plays a significant role in influencing our social lives, whether publicly in the area of politics, or privately, in terms of personal usage and communication. Learning about creative acts in various geographical locations depends heavily on journalistic reporting during the pandemic, which is especially important in light of the limited ability to travel while in quarantine. In fact, following the Center for Disease Control guidelines during the pandemic to limit social gatherings and travel has made it very important to find new ways to stay connected to others. As a result, electronic news sources have become extremely valuable for understanding news in various communities and across the nation.

The purpose of this media analysis is to understand how individuals, groups, and communities demonstrated creative actions in response to the constraints of the COVID-19 pandemic. We selected a media analysis approach (Altheide and Schneider, 2013) to answer the research question, “How are people using creativity in various communities in response to the pandemic?” The study examines creative responses to the COVID-19 crisis as documented by two newspapers of record *The Los Angeles Times* and *The New York Times*.

## Method

### Procedure

Using the keywords “creativity” and “COVID,” we collected *New York Times* and *Los Angeles Times* articles published between March 17, 2020 and August 6, 2020. Both newspapers are included in the top ten newspapers in the United States in terms of circulation, and each have a circulation above 400,000. These newspapers were selected to represent the current events occurring in the United States during the early days of the COVID-19 pandemic. The purpose of collecting articles on the east coast and west coast was to contrast reactions according to the differing chronologies and manifestations of the disease on each coast. New York was a hotspot for COVID-19 early in the pandemic compared to Los Angeles where the disease appeared early, but the rise in cases was slower. Articles were gathered from these two newspapers using the search function available to subscribers through their website. Articles that described a “creative action” in response to COVID-19 were retained for analysis. Media analysis (Altheide and Schneider, 2013) is a form of social science research that analyses published accounts of social life in public forums like newspapers, websites, social media, and/or blogs.

### Coding

Media articles that contained examples of creative action during the pandemic were saved for further analysis in the data collection stage. The unit of analysis was specified using the terms “creative” as a description of an action, not merely in an unrelated remark, and “COVID-19.” Articles that did not meet this criterion were discarded. Fifty articles were kept for analysis. “Creative actions” ranged from traditionally conceived artistic pursuits such as painting, photography, and playing music to actions of creative problem solving where acts were described as “novel and appropriate” (Sternberg and Lubart, 1999).

In the first round of coding, the research team reviewed sample media articles together to arrive at a common definition of “creative action.” After an initial process of scoping the news articles for themes, a codebook was generated that listed prominent themes related to creativity during COVID-19 (Saldaña, 2014, 2015). Geertz’s (1973) definition of culture and principles of socio-cultural theory were used to inform the categorization of creative actions into themes. The codebook contained the number and name of the code, the definition of each code and examples. Three independent coders then read each article line by line to determine the presence of each code. The codes were: group or collaborative creativity, creativity as relational, power dynamics within creativity/culture, individuals’ relationship to creativity/creative process, adaptation of creative domains, traditional representations of creativity being renewed or continued, traditional evaluations of creativity challenged or reshaped, creative adaptations to societal ritual or routine, the negative impacts on creativity, and creativity as social justice.

To address the validity and reliability of the codes, coding occurred iteratively. Coding was discussed in team meetings and coding was examined across team members (Merriam, 2009). The codebook was shared and updated frequently to ensure an audit trail, or detailed account of the procedures and coding decisions (Merriam, 2009). When new concepts emerged, the team discussed them. Three codes “creativity for social justice,” “negative impact of creativity,” and “creative resilience” were added during the discussions. Then, the research team re-read the media articles and coded for these three new codes and updated the data, attesting to adequate engagement in the data analysis (Merriam, 2009). The codebook was shared again with updated data, to make sure the codes were applied consistently to the articles across the team members.

In the second round of coding, creative actions were organized into the following two major themes: renewal and continuity in creative communities and the multidimensionality of creativity. The data analysis strategy of theoretical propositions (Yin, 2018) was used to ground the codes within a socio-cultural perspective of creativity, which posits that creativity is a culturally situated action wherein “(creativity) uses the signs and tools made available by… (culture) to produce new cultural resources that go on to facilitate future creative acts” (Glãveanu et al., 2019, pp. 2).

## Results

First, we provide an overview of the codes across all 50 articles to show the numerical range of the results, and the salience of certain codes. Next, we explain our two integrated themes and list the codes related to each theme. Each code is explained through excerpts from the articles, to show how the codes connect to the data. We highlight the conceptual diversity (Merriam, 2009) of the codes, representing the heterogeneity of creative actions.

### Distribution of Codes

The codes listed in Table 1 represent an open coding process influenced by a social-cultural perspective of creativity (Glãveanu et al., 2019). The total number of instances of creative actions coded across all 50 articles was 959 (Table 1). The average number of coded instances of each code was 88 instances. The code that emerged most frequently was *Creative adaptations of social rituals or routines*, with a total of 153 instances across all 50 articles. The second most prolific code was *Adaptation of creative domains during COVID-19*, with 141 total instances across all 50 articles. The frequency of these two codes emphasizes the salience of these two codes in the articles.

**TABLE 1:** Typology of creative actions (*N* = 11) distributed across media articles.

		**Multi-Dimensionality of Creativity**					**Renewal and Continuity in Creative Communities**						
			
	**Code**	**Group creativity**	**Creativity as relational**	**Power dynamics**	**Individual creativity**	**Creativity as resilience**	**Adaptation of creative domains**	**Tradition continued or renewed**	**Tradition challenged or reshaped**	**Adaptation to ritual or routine**	**Negative impact on creativity**	**Creativity as social justice**	
	***n***	**101**	**103**	**38**	**94**	**65**	**141**	**52**	**52**	**153**	**110**	**50**	**959**
	**%**	**11%**	**11%**	**4%**	**10%**	**7%**	**15%**	**5%**	**5%**	**16%**	**12%**	**5%**	

**Author**	**Date**												***n***

Daswani	21-March-2020	1	2			1	1		2		4		11
LA Times	21-March-2020	1	1									1	3
Burch, 2020	21-March-2020	3	3		5	3	1	2	1	1	1		20
McNulty	22-March-2020				1				1				2
Klass, 2020	24-March-2020					1							1
Wheeler	26-March-2020			9	1	1			3		10	1	25
Mittler, 2020	01-April-2020			1	3		2				5	4	15
Rodell	02-April-2020		4				1		2	5			12
Halper and Hook, 2020	03-April-2020	8		5						6		2	21
Roberts, 2020	08-April-2020	1			1			1			1	1	5
Paton and Testa	09-April-2020						5	2	1	3	4		15
Modak, 2020	13-April-2020	1	1		2	1		1		1	2	2	11
Lee, 2020	14-April-2020				1	1						1	3
Kaufman	14-April-2020	10	12		5	7	10						44
Kelleher	16-April-2020		1			3	1	1	3	1	2		12
NYT Staff	21-April-2020	6	8		12	11				5	5	3	50
Garcia	23-April-2020		2		1	3	9		3		4		22
Barbaro, 2020	24-April-2020	1			1	1				2			5
NYT Staff	02-May-2020	12	8	4	1	7	8	6	1	4	1		52
Richtel	04-May-2020						2		3	3	2		10
Pineda	10-May-2020	2	1		1	2	9		4	1	13		33
Alexander	12-May-2020	21	23							23			67
Marshall, 2020	22-May-2020	10	7	2	10	3	3	5	1	1	1		43
Castilo, 2020	23-May-2020									1	1		2
Wilson	24-May-2020		2										2
Matar, 2020	24-May-2020	4	2	3	7	6	2	3	3		3	5	38
Powell and Eligon, 2020	24-May-2020	1		3						3	3	5	15
Friedman	25-May-2020		1	2	1	1	5	1	2	1			14
Hars	26-May-2020				1		3			4	2	1	11
Miranda	28-May-2020			1	2		30			34	4		71
Cerveris	31-May-2020	1	1	2		1		2	3			7	17
Martens, 2020	01-June-2020		1		1	1	2	1					6
Abkowitz, 2020	02-June-2020	1	1		1		3	3	1	4	1		15
Whip, 2020	04-June-2020	2	1	1		1		1	1	1			8
Lai, 2020	24-June-2020		5		5		3	3	1	6	3	3	29
Pineda	02-July-2020				6	1		6	1	1	4	1	20
Austen	03-July-2020			1						3			4
Swed	16-July-2020				4	2	4		1	6	1	2	20
Clendenin	17-July-2020	5					5	5					15
Easter, 2020	20-July-2020	5	4	2	6	2	3	4	1	3	5		35
McNulty	20-July-2020	1		1			3	1	6		2	9	23
Saad, 2020	21-July-2020		11			2					1		14
Friedman	23-July-2020				5	1	6			10			22
Chang, 2020	23-July-2020			1				2	2		12		17
McNamara	24-July-2020				1					1			2
McNamara	30-July-2020				8	1	5			5	3		22
Arellano	30-July-2020	3								3			6
Battaglio, 2020	31-July-2020						2	2		6	3		13
Pineda	31-July-2200	1	1		1		8		4				15
Tschorn	26-August-2020					1	5		1	5	7	2	21

On average, each code appeared at least once in 24 out of 50 articles. The code with the lowest number of appearances was *Power dynamics within creativity*, which appeared in only 15 of the 50 articles used in analysis. The code that appeared in the most articles was *Creative adaptations of societal rituals or routines*, recognized in 30 one out of 50 articles. The second most commonly appearing code was *Negative impact on creativity*. This code appeared in 30 out of 50 total articles. *Adaptation to ritual or routines* and *Negative impact on creativity* appeared in more than twice as many articles as *Power dynamics within creativity*. While the code *Adaptations of creative domains* recorded more specific instances than *Negative impact on creativity* (141:110), the code *Negative impact on creativity* appeared in more articles (30:28).

The articles supplied from the *Los Angeles Times* and the *New York Times* provided a great diversity of examples of creativity during this COVID-19 pandemic. The following paragraphs highlight the various manifestations of these codes as they appeared in articles.

### The Multidimensionality of Culture Within Creative Communities

The analysis of the data found a wide range of creative actions. The first integrated theme within the results brings together these examples as evidence of the multidimensionality of culture within creative communities. Codes within this theme include group or collaborative creativity, creativity as relational, power dynamics of creativity, individual creativity, and creativity as resilience. The following paragraphs elaborate on the nature of these codes and provide relevant examples from the data.

#### Group or Collaborative Creativity

Twenty three of the 50 articles analyzed in this study provided at least one example of groups of individuals responding to COVID-19 by being creative together. In the New York Times article “Quarantined and Engaged: They said Yes!” Charanna Alexander documents the creative ways that couples adapted their marriage proposals because of COVID-19 quarantine regulations. One of these couples described how out-maneuvering the constraints of the quarantine led to the following scene, “200 of our closest family and friends muted, waiting on Zoom just out of my sight. She asked me to marry her, and then turned me around to see everyone holding up signs that read *Love is Not Canceled*” (May 4, Alexander, 2020).

Another elaborate example of creative collaboration occurred in an assisted living community. Members of this community transformed their own “news channel” into a pandemic response. “When coronavirus began sweeping through southern California… channel 22 pivoted to live-streaming 3 days a week, 8 h a day” (April 14, Kaufman, 2020). Different members of the community pitched in to create new shows and promote safety routines to control for the spread of the virus. They adapted something they had created into something new, something they needed because of COVID-19, “We are providing a bit of a life raft for people who feel like they are on their own” (April 14, Kaufman, 2020). Shows produced by members of the community include a “poetry show, a writing club called the “gray quill society,” and the “original foodie” as well as Digital Angel Cards…” (April 14, Kaufman, 2020). These angel cards allow family members to send images, videos, or text to loved ones who they can’t visit because of quarantine restrictions.

#### Creativity as Relational

Throughout the 50 articles, numerous examples of individuals and groups acting creatively to fortify relationships and maintain social connection across new social barriers were found. The social-distancing measures put in place to mitigate the spread of COVID-19 have made it so that people have had to find novel ways of interacting and connecting. For instance, when coronavirus confined Australians to their homes, a neighborhood in Melbourne began holding regular “isolation happy hours from across the laneway” (April 2, Rodell, 2020). Other examples include neighbors in the United States using an app to reach out and help one another, choirs meeting online to hold musical movie nights, and Italians singing to one another out of their windows (New York Times, 2020a). The constraints of the pandemic have also inspired people to connect in different ways with relatives at home, using creativity as a conduit; for instance, a quarantined actress was surprised and delighted to become an audience member as her two young children performed plays on their big coffee table at home (May 24, Wilson, 2020). These examples support the notion that creativity is socially situated, where creative actions are given meaning within a greater social context. The confines of quarantine have highlighted instances of creativity that run contrary to its common association with individualism, where people are seen reaching out creatively to bolster relationships and work toward a collective good.

#### Power Dynamics Within Culture

One of the less frequently explored themes across all 50 articles was power dynamics within creativity. Ian Wheeler, the co-founder of Partisan Records and Talkhouse, described the impact of the pandemic as revealing “how poorly designed our infrastructure is for supporting artists” (March 26, New York Times, 2020b; Wheeler, 2020). Mr. Wheeler points out that many artists rely on live performances for income and the restrictions imposed by the government sanctioned quarantine has compromised their economic security. “We put a lot of pressure on creatives to make the things that make us feel better, and we sometimes forget they may be suffering, too” (March 26, Wheeler, 2020). While it may seem obvious to point out that money and power dynamics are intertwined, COVID-19 has emphasized the powerful role of finances in the lives of professional artists. “Too many people responsible for creating… have been left in limbo for the foreseeable future, unsure of when and how their next paycheck will materialize (March 26, Wheeler, 2020). In their collection of stories about the impact of the pandemic on photographers, one artist describes how her mother could no longer support herself after decades of performing as a professional clown, “I woke up one morning, and saw my mother crying. She had been trying to file for unemployment. This was heartbreaking” (April 21, New York Times, 2020a,b). While public health research shows the quarantine is necessary, there are undeniable power dynamics involved when government restrictions suspend individuals’ ability to perform their art. Recognizing these power dynamics may be necessary in order to address what Mr. Wheeler says is a “poorly designed infrastructure” (March, 26, Wheeler, 2020).

#### Individual Creative Processes

One of the most commonly recognized phenomena across all 50 articles was COVID-19’s impact on individual creativity. Articles from both sources chronicle the impact of quarantine on artists in the following domains: photography, dance, film, music, and others (New York Times, 2020b). There were two specific kinds of responses to the pandemic regarding individuals’ relationship to creativity: altering the creative vision and altering the creative process. An article about Puerto-Rican sculptor Daniel Lind-Ramos documents how the artist has incorporated the crisis into his creative vision, “Watching the coronavirus crisis take hold, Mr. Lind-Ramos’s notebook filled with carnival characters and their accessories, augmented by medical and hygienic references… the sketches are blueprints for new sculptures” (April 1, Mittler, 2020). While Daniel Lind-Ramos has shifted his creative inspiration due to the pandemic, other artists have adapted their means of sharing their work.

Dance choreographer Mark Morris shifted from working in his dance studio to creating and showing his “video dances” online (May 26, Haras, 2020). Using Zoom and Final Cut Pro, the renowned choreographer is able to apply his craft through a new medium. Mark Morris isn’t alone. Beyond dance, the articles from this analysis provided examples of fashion (July 23, Friedman, 2020a), photography (July 17, Clendenin, 2020), music (July 16, Swed, 2020), theater (July 20, McNulty, 2020b), among other art forms all moving toward integrating online performances and thereby requiring creative changes within the relationship of artist to the creative process.

#### Creativity as Resilience

A common tone that wove throughout many of the articles was one of general anxiety (Los Angeles Times, 2020). There was a prevailing gloominess about the situation and people were feeling trepidation of what was yet to come. Despite this tone, various articles contained examples where individuals used creativity as a form of resilience in the face of sadness, trauma, and anxiety. For example, in response to the death of a well-known singer-songwriter due to complications of coronavirus, a choir rehearsed for over a month to put together a Zoom video of one of his songs. One of the choir members said, “it gave (her) something to focus on that didn’t involve all the desperation” (April 23, Garcia, 2020; McNulty, 2020a). A different example of a distraction from the anxiety of the pandemic was a popular social media challenge where people posted pictures of themselves re-creating works of fine art. People said that engaging in this creative challenge was “a way to lighten things up,” “put things in perspective,” and “hopefully make people laugh” (April 17, Kelleher, 2020). There was also an example of professional clothing designers channeling the mass hysteria associated with the pandemic to create a new collection that featured cultural memes from the pandemic such as rolls of toilet paper and hands being washed (March 21, Daswani, 2020). These examples show people using creativity as a mechanism of resilience when facing bleak circumstances. These instances underscore how creativity can give meaning and bring joy to human existence (Glãveanu et al., 2019).

### Renewal and Continuity in Creative Communities

Various articles documented “creative actions” that renewed cultural experience and preserved the continuity of tradition. Renewal and continuity also refer to the shared materials, traditions, and institutions utilized in everyday creativity as described by Tanggaard’s (2013) theory of socio-material creativity. In these articles, creativity did not occur only in the isolation of the individual experience. Creative actions within this theme provided examples of adaptation of creative domains, traditional representations of creativity being renewed, traditional evaluations of creativity being challenged, creative adaptations to ritual or routine, negative impacts on creativity, and creativity for social justice. These results demonstrate how communities brought cultural traditions and past creative actions forward and used them in new, novel and appropriate creative actions.

#### Adaptation of Creative Domains

Several articles documented COVID-19 as a disrupting factor to creative industries. In their article for the *New York Times*, Elizabeth Paton and Jessica Testa found that limitations on photo shoots caused some publications to alter their means of production. “In upcoming issues of *GQ*, some subjects are photographing themselves at home with their own devices – instant cameras and iPhones – in consultation with photographers; others will be represented without photography. In some cases, illustrations will replace traditional portraits” (Paton and Testa, 2020). Featuring illustrations rather than portrait photography could be considered an homage to fashion magazines published at the beginning of the 20th century before photography replaced illustration as the primary visual trope for marketing fashion. The influence of the past is not the only creative inspiration according to the authors, “From a creative standpoint, editors and publishers said they are taking advantage of the chaos. It’s an excuse to try new things.” Fashion is perhaps the most predictable example of continuity and renewal within a creative field.

Interviews with architecture and design experts from around the country about the effects of COVID-19 on the field revealed inherent difficulties around social distancing to prevent the spread of the coronavirus- “…one of architecture’s core purposes – creating containers that bring people together – seems almost inconceivable” (Miranda, 2020). The types of offices that made workers feel safe and productive may revert back to offices with more restrained contact between employees, with sanitation duties considered a vital part of employee responsibility. “In the early 20th century, concerns about tuberculosis and sanitation helped shape Modernism.” The stage is set for viral contagiousness to return as primary design consideration. “If we don’t get a vaccine… what does that mean in terms of physical space? What do you do with a doorknob?” asks Hernan Diaz Alonso, director of Southern California Institute of Architecture (Miranda, 2020). The context of COVID-19 has altered the constraints of the creative process in less obvious ways as well. “The design of new buildings has largely halted, threatening the economic stability of many firms” (Miranda, 2020).

#### Traditional Representations of Creativity Being Renewed or Continued

Creative actions helped to preserve traditional cultural productions during the COVID-19 quarantine. Articles in both the *Los Angeles Times* and *New York Times* highlighted schools altering theater programming due to the COVID-19 quarantine (Goldberg, 2020, Pineda, 2020b). For example, students at Los Angeles County High School for the Arts faced cancelations of performances and rehearsals. The principal of the school, John Lawler, described how students and teachers adapted, saying “people are… tapped into their *creative spirit* because they’re coming up with ways to perform and rehearse, and to collaborate, even from these remote locations” [italics added] (Pineda, 2020b). The production of “West Side Story” at LACHSA had a budget of eighty thousand dollars, over 50 cast members, and 30 orchestra students. The creative process of producing this theater classic now includes a new wrinkle: determining how to film and broadcast the performance online. The adjustment is not only technical–the students must also adjust to performing without an audience (Pineda, 2020b).

Social media provided a new way to appreciate traditional artwork. Art recreation is an Instagram challenge that requires participants to create a tableaux vivant of a famous work of art, take a picture, and share the results online. “Tens of thousands of re-creations appear under the hashtags #mettwinning, #betweenartandquarantine, and #gettymuseumchallenge. Some have been made by arts professionals, but many of them are the skillful work of amateurs (Kelleher, 2020). Art re-creations can be done by individuals or groups, and the idea even works for online classrooms to get students in touch with art when they cannot visit a museum. “At a time when museums are closed, galleries have shuttered and art education has largely moved online, these images have formed a living archive of creativity in isolation” (Kelleher, 2020).

#### Traditional Evaluations of Creativity Challenged

Several articles featured individuals applying a critical lens to traditional creative domains. One such interview in the Los Angeles times investigated how COVID-19 has challenged the ways creativity is evaluated within theater arts. This was one example of artists advising other artists to use this “opportunity” (COVID-19) to challenge what producers expect, and what audiences should anticipate from their creative institutions (July 20, McNulty, 2020b). Michael Cerveris (2020), May 31 wrote a letter to the Los Angeles Times giving advice to young theater artists saying, “I do see in this dark, chaotic time reasons for great optimism. When the rules no longer apply, we can write new rules.” He imagines a theater with new priorities that “values creativity, simplicity, and ingenuity over lavish producing, marketing, self-congratulating, and selling.” In his brief column of advice, he challenges the pervasive model of evaluating the creative worth of a production through the lens of profits and notoriety.

COVID-19 has altered expectations of creativity, not only by constraining traditional expressions, but through diversifying the needs of our communities. Few of us will remember the option of contact tracing as a career choice in high school. According to Dorany Pineda of the LA Times, contract tracing requires workers who are organized, meticulous, and curious, all skills possessed by a unique community of existing workers: librarians. Her article demonstrates how several librarians adapted their skills to provide a new and unique service to their community (July 31, Pineda, 2020a). This creative act of repurposing a trained skill, to be used in new ways, for unforeseen purposes, demonstrates how creativity can be found outside traditional domains.

#### Creative Adaptations to Ritual or Routine

The articles reviewed revealed many examples of creative adaptations to societal rituals and routines. The code *Creative Adaptations to Ritual and Routine* was identified most frequently across the 50 articles. The ubiquity of this code reflects the ways in which people have reacted adaptively, in small and big ways, across various parts of their lives. The prevalence demonstrates how “creativity does not only lead to societal progress through notable inventions and discoveries, it does so also (if not primarily) by changing the way people relate to the world, to others, and to themselves, making them more flexible, more open to the new” (Glãveanu et al., 2019). Due to social distancing protocols, many societal rituals have been waylaid by the pandemic such as birthday parties, graduations, funerals, and holidays (Wantanabe, 2020). Even routine behaviors, such as going to work or traveling, have undergone creative adaptations. One article chronicles a family’s organization of a socially distant, outdoor memorial to pay homage to a beloved family and community member (July 30, Arellano, 2020). In another creative adaptation to social ritual, the food section at a local grocery store included a thoughtful package about celebrating Passover and Ramadan under the conditions of the pandemic (April 2, Rodell, 2020). Just as churches altered their Sunday worship services during the flu of 1912 (Smith, 2020), traditional Easter celebrations have been adapted or canceled because of COVID-19 (Dias, 2020; Parvini, 2020). People have also had to contend with changes to daily routines as people have adapted to working from home or changes in office set-up (May 4, Richtel, 2020). On a larger scale, a massive behavioral and cultural shift is happening as people are adapting to wearing face masks in their daily lives (July 3, Austen, 2020). These changes to ritual and routine have not necessarily been met with enthusiasm, and in some cases have been met with active resistance.

#### Negative Impact on Creativity

While the 50 articles sampled in this study articulated the myriad ways that people supported each other as they confronted and endured the effects of the pandemic, an unexpected theme was the negative impact of the pandemic on creativity. Many articles included strong statements describing the negative impact of COVID-19 on creativity. These statements referenced job loss, financial loss, anxiety, difficulty, and uncertainty. Being in lock-down for unanticipated periods of time isolated people and instilled deep reflection. As a result, people articulated a range of consequences, from the concepts of reinvention to transformation to blatantly stymying creativity. For example, some people felt “creativity was being jeopardized” (Michele in Friedman, 2020a, pp. 17) and the weight of these negative consequences were “taxing” (Tschorn, 2020). Art students found it heart-breaking to perform online instead of in person, for example. Actor Uzo Adoba described the psychological toll of the negative impact on creativity (McNamara, 2020b, pp. 4–5):

“The anxiety is real… you can see it everywhere. At a grocery store, at a pharmacy, the number of times people look down at the sticker to make sure they’re six feet apart, the shock you feel when you see someone not wearing a mask. Little patterns of behavior.”

#### Creativity for Social Justice

Some people described a sense of gaining or finding a new “voice” despite the negative impacts of the pandemic mentioned above. In two examples, members of marginalized communities discussed ways they struggled to achieve acknowledgment in three interrelated areas: immigration, citizenship and economic opportunity. In one story about drive thru centers that were set up for citizenship tests in order to manage social distancing, one person commented, “It feels good to actually have a voice now. It’s a huge blessing.” Thus, despite anti-immigration sentiments, the articles showed how people persevered to see their dream of becoming an American citizen a reality. In another story, Pineda (2020) described ways that people have created micro-economies of selling various foods on the street. Even though he had struggled to work on various farms in Mexico to support his family, he was not able to achieve economic justice until he traveled to the United States to set up his own business selling ice cream. While the work remains hard, Mr. Rios states: “It’s fun and it’s distracting… and I’m happy doing what I do,” (Pineda, 2020c, pp. 17).

## Conclusion and Discussion

In conclusion, this media analysis study of 50 articles from the *New York Times* and *Los Angeles Times* in the summer and spring of 2020 revealed how creative actions renewed and sustained cultural institutions while also demonstrating the profound multidimensional aspects of culture in creativity. While the COVID-19 pandemic forced social isolation on communities, the results of this study highlight new communal and culturally situated creative responses to the quarantine. Creative actions with cultural and environmental affordances supports the socio-cultural perspective of creativity (Glãveanu et al., 2019) in terms of unearthing the themes of group and collaborative creativity, relational creativity, adaptations to creative domains, creative traditions being continued and challenged, and changes to societal ritual and routine found within the 50 articles resulted in two implications for creativity research. Additional themes of power dynamics within creativity, individual’s relationship to the creative process, creativity as resilience, the negative impact of COVID on creativity and creativity as social justice also emerged from the articles. Below, we discuss two implications of this study, limitations and suggestions for further research.

The first implication was that creativity was an observable, communal, cultural response to the COVID-19 crisis. This finding is consistent with a socio-cultural view of creativity (Glãveanu et al., 2019). As seen in the examples of schools, orchestras, and churches, the pandemic revealed itself as a new manifestation of “affordance” (Glǎveanu, 2013) that inspired creative adaptations to established traditions. For some creators, the pandemic provided the inspiration behind new creative actions. Moreover, there were myriad examples of individuals acting creatively collectively to approach issues brought on by pandemic and resulting quarantine. Creativity is at all times relational (Glãveanu et al., 2019) as was exemplified by the ways in which groups adapted existing social domains and came up with novel ways of interacting. Examples of people using creativity as resilience during the pandemic was an unexpected code that emerged from the articles. Research, mostly within the field of Art Therapy has not fully explored how individuals use creativity as a form of resilience and coping (Sternberg et al., 2019). Other researchers, examining the implications of the pandemic, have observed creativity as a form of resilience (Yang, 2020) and more research may be forthcoming. However, creativity as a communal act of resilience remains largely under-explored. The observable nature of COVID-19 as a cultural phenomenon that inspired creative action led to an expanded view of how cultural resources interact with creativity at the communal and individual level.

The second implication of the study expands on the “Five A’s” category of “affordances” by demonstrating how cultural resources work as dynamic constraints that influence how groups and individuals behave creatively. Access to cultural resources due to COVID-19 functioned as a constraint, in some instances facilitating and other instances hindering creative actions. Emergent themes that demonstrated this effect were the negative influence of COVID-19 on creativity and creativity as an expression of social justice. Anxiety, social isolation, lack of financial support and stress about one’s health were all negative impacts on creative action triggered by COVID-19. While some established artists were able to adjust creatively to new circumstances, others lost their primary source of income. Some artists found self-quarantine to be an empowering decision laden with creative opportunity. For other artists, with less access to resources, the quarantine forced them into personal and economic vulnerability. More broadly, the impact of the pandemic was exacerbated within communities who already experience unequal rights in society as it was harder for them to access the creative, cultural resources needed to achieve their goals.

The COVID-19 pandemic and quarantine also functioned as a constraint for the researchers of this study. One limitation forced on the data collection process was the necessity to rely on journalism rather than direct human observation due to the ban on face to face research with human subjects. The use of media articles provided a lens to study a global phenomenon via news media reporting on the pandemic. However, future research that includes a wider range of news sources, dates and quantity of articles would provide a larger scope of transferability. Alternatively, direct and focused observation of individuals would have provided the opportunity for richer description. A longer study could follow up with each article or reporter regarding the specific circumstances and even conduct follow up interviews with the subjects of the articles. Time constraints, financial resources and the risks of COVID-19 all influenced the design and methods used in this study.

Based on this discussion, future creativity research is needed which investigates the nature of diverse cultural resources in various communities, as well as access to cultural resources that serve as constraints for the development of creative identity. Cultural resources, defined as any product derived from the use of cultural artifacts like language and symbols, are essential for any creative action (Glãveanu et al., 2019). Research on creativity conducted through a socio-cultural lens allows for a broader perspective that can be used to examine power dynamics and social justice issues within creativity. The socio-cultural theory of creativity inspires a contemporary area for investigation within the field of creativity research. Since the initial period of data collection on the news articles, the growing call for social justice and racial equality in the United States places an urgent emphasis on examining creativity in marginalized communities in new, untapped ways. The multidimensionality of creativity represented in this study provides a new example of how creative actions that might otherwise be categorized into different fields of creativity research can be analyzed and discussed using a socio-cultural perspective of creativity. This study, along with other creativity research that makes use of a socio-cultural perspective, should further contextualize the experiences of creative actors with an increasingly diverse, multidimensional vision of creative action.

## Data Availability Statement

The original contributions presented in the study are included in the article/supplementary material, further inquiries can be directed to the corresponding author/s.

## Author Contributions

All authors listed have made a substantial, direct and intellectual contribution to the work, and approved it for publication.

## Conflict of Interest

The authors declare that the research was conducted in the absence of any commercial or financial relationships that could be construed as a potential conflict of interest.

## References

[B1] Abkowitz A. (2020). With caution and creativity, day cares prepare to reopen. Isolation rooms, crib dividers and designated school shoes are just a few examples of the health measures that child care centers are taking. *New York Times.*

[B2] Alexander C. (2020). Quarantined and engaged: they said ‘020!’ The coronavirus didn’t stop these couples from celebrating their love with fun, creative and romantic proposals. *New York Times.*

[B3] Altheide D. L. Schneider C. J. (2013). *Qualitative Media Analysis*, 2nd Edn. Thousand Oaks, CA: Sage.

[B4] Arellano G. (2020). In San Bernardino, a legendary waitress gets a COVID-19-safe memorial, with tacos to go. *Los Angeles Times.*

[B5] Austen I. (2020). Sorting out Canada’s patchwork of face mask rules, without general directives from federal and provincial governments, local leaders have been left setting mask directives. *New York Times.*

[B6] Bakhtin M. M. (1994). *The Bakhtin Reader: Selected Writings of Bakhtin, Medvedev, and Voloshinov.* London: Hodder Education.

[B7] Barbaro M. (2020). A funeral, reinvented for the pandemic; we set out to tell the story of how grieving is changing in this moment. *New York Times.*

[B8] Battaglio S. (2020). The COVID-19 pandemic is forcing networks to reinvent TV election coverage. *Los Angeles Times.*

[B9] Burch A. (2020). How coronavirus-weary Americans are seeking joy. The spread of the virus has cut Americans off from each other, but some are using the opportunity to enjoy more simple pleasures. *New York Times.*

[B10] Castilo J. (2020). Dodgers have small window to get full effect of draft. *Los Angeles Times.*

[B11] Cerveris M. (2020). Letter to the editor. *Los Angeles Times.*

[B12] Chang J. (2020). Movie theaters are still shut down — and with them, the full power of challenging cinema. *Los Angeles Times.*

[B13] Clendenin J. (2020). Ask a reporter: photographer Jay Clendenin on making celebrity portraits during COVID-19. *Los Angeles Times.*

[B14] Csikszentmihalyi M. Rochbert-Halton E. (1981). *The Meaning of Things: Domestic Symbols and the Self*. Cambridge, MA: Cambridge University Press.

[B15] Csikszentmihalyi M. (1998). “Implications of a systems perspective for the study of creativity,” in *Handbook of Creativity*, ed. Sternberg R. (Cambridge: Cambridge University Press), 313–335. 10.1017/cbo9780511807916.018

[B16] Daswani K. (2020). L.A. Brand creates coronavirus-inspired collection to benefit creatives who’ve lost jobs. *Los Angeles Times.*

[B17] Dewey J. (1934). *Art as Experience.* New York, NY: Penguin.

[B18] Dias E. (2020). A sunday without church: in crisis, a nation asks, what is community? *New York Times.*

[B19] Easter M. (2020). Here’s why COVID-19 has made arts education so problematic. *Los Angeles Times.*

[B20] Friedman V. (2020a). Angels and artisans and lots of ambition at Valentino and Dior, the last of the pandemic-designed digital shows. *New York Times.*

[B21] Friedman V. (2020b). Gucci says fashion shows should never be the same. The Italian brand is reducing its runway schedule. Your instagram is about to change. *New York Times.*

[B22] Garcia S. E. (2020). How to stay creative while stuck at home The pandemic has disrupted the creative lives of everyday Americans who used to spend untold hours together singing, dancing and making art. But creativity finds a way. *New York Times.*

[B23] Geertz C. (1973). *The Interpretation of Cultures: Selected Essays.* New York, NY: Basic Books.

[B24] Glaveanu V. Lubart T. Bonnardel N. Botella M. De Biaisi P. M. Desainte-Catherine M. (2013). Creativity as action: findings from five creative domains. *Front. Psychol.* 4:176. 10.3389/fpsyg.2013.00176 23596431 PMC3627136

[B25] Glǎveanu V. P. (2013). Rewriting the language of creativity: the five A’s framework. *Rev. Gen. Psychol.* 17 69–81. 10.1037/a0029528

[B26] Glǎveanu V. P. (2015). Creativity as a sociocultural act. *J. Creat. Behav.* 49 165–180. 10.1002/jocb.94

[B27] Glãveanu V. P. Hanchett Hanson M. Baer J. Barbot B. Clapp E. P. Corazza G. E. (2019). Advancing creativity theory and research: a socio−cultural manifesto. *J. Creat. Behav.* 54 741–745. 10.1002/jocb.395

[B28] Goldberg M. (2020). Remote school is a nightmare. Few in power care. *New York Times.*

[B29] Halper E. Hook J. (2020). No rallies? Activists find voters in online worlds; the pandemic pushes politicking into video games and could force a virtual convention. *Los Angeles Times.*

[B30] Haras M. (2020). The pragmatist’s progress: Mark Morris adapts to creating online, “I never had an interest in technology before,” said the choreographer, whose first batch of online dances will stream this week. *New York Times.*

[B31] Jaramillo J. A. (1996). Vygotsky’s sociocultural theory and contributions to the development of constructivist curricula. *Education* 133–141.

[B32] Kaufman A. (2020). Isolated but keeping in the loop. Residents of showbiz retirement home tune in to own TV channel to stay connected. *Los Angeles Times.*

[B33] Kelleher K. (2020). Art recreation is the only good instagram challenge. Around the world, people are posing as famous portraits with toilet paper, bedsheets, drawn-on unibrows, and did we mention toilet paper? *New York Times.*

[B34] Klass P. (2020). Getting through, making memories and being the grown-ups. You can’t change the world, but you can help shape the way your children experience this and remember it. And you will. You’re the person they need. *New York Times.*

[B35] Lai S. (2020). In the COVID-19 era, they become U.S. citizens in a drive-through. *Los Angeles Times.*

[B36] Lee A. (2020). The little voice inside. Sara Bareilles’ series rethinks the villain of music biz TV shows; meet self-doubt. *Los Angeles Times.*

[B37] Los Angeles Times (2020). The big one, but not what we envisioned. *Los Angeles Times.*

[B38] Marshall A. (2020). Creating an exhibition of Britain’s lockdown dreams. The artist Grayson Perry talks about his latest TV series, “Grayson’s Art Club,” which showcases art made by a British public trapped at home. *New York Times.*

[B39] Martens T. (2020). The scariest film? This Instagram game. *Los Angeles Times.*

[B40] Matar H. (2020). Something happens when you fall two artworks that ask the question: what world will we find on the other side of this? *New York Times.*

[B41] McNamara M. (2020a). Column: Sherlock Holmes and Mary Russell are getting me through COVID-19. They can help you too. *Los Angeles Times.*

[B42] McNamara M. (2020b). Column: how Emmy nominee Uzo Aduba, Ann Dowd and more actors stay creative amid COVID-19. *Los Angeles Times.*

[B43] McNulty C. (2020a). Mastering the art of doing nothing; why now is the ideal time to settle into boredom. *Los Angeles Times.*

[B44] McNulty C. (2020b). How should L.A.’s legacy theaters change after COVID-19? Well, for starters. *Los Angeles Times.*

[B45] Merriam S. B. (2009). “Qualitative data analysis,” in *Qualitative Research**: A Guide to Design and Implementation*, (San Francisco, CA: Jossey-Bass), 169–207.

[B46] Miranda C. (2020). How should L.A. be redesigned for coronavirus? Are doorknobs out? We asked the experts. *Los Angeles Times.*

[B47] Mittler S. (2020). This Puerto Rican sculptor meets disaster with spirit watching the coronavirus crisis take hold, Daniel Lind-Ramos, a powerful storyteller, filled notebooks with carnival, medical and spiritual imagery. *New York Times.*

[B48] Modak S. (2020). Missives from my locked-down friends, from Siberia to Samarkand. The people I met as the 52 places traveler were suddenly just as close as my friends down the street, so I reached out to my global community. *New York Times.*

[B49] Moran S. John-Steiner V. Sawyer R. (2003). “Creativity in the making: Vygotsky’s contemporary contribution to the dialectic of development and creativity,” in *Creativity and Development*, eds Sawyer R. K. JohnSteiner V. Moran S. Sternberg R. J. Feldman D. H. Gardner H. (New York, NY: Oxford University Press), 61–90. 10.1093/acprof:oso/9780195149005.003.0003

[B50] New York Times (2020a). As restrictions lift, countries brace for a new reality. As lockdowns begin to ease for millions of Europeans, Italy reports a spike in deaths. Migrants face roundups in Malaysia and surging infections in Singapore’s dormitories. *New York Times.*

[B51] New York Times (2020b). Still lives; in this unnatural state of isolation, photographers show us the things that bind. *New York Times.*

[B52] Parvini S. (2020). Faithful find creative ways to celebrate Easter together, separately. *Los Angeles Times.*

[B53] Paton E. Testa J. (2020). What’s the point of a fashion magazine now? *New York Times.*

[B54] Pineda D. (2020a). As COVID-19 cases surge, L.A. librarians join the ranks of contact tracers. *Los Angeles Times.*

[B55] Pineda D. (2020b). High school performing arts were shut down by coronavirus. Will it derail students’ careers? *Los Angeles Times.*

[B56] Pineda D. (2020c). L.A. ice cream vendor adapts to life in a COVID-19 world. *Los Angeles Times.* Pineda D. (2020). As COVID-19 cases surge, L.A. librarians join the ranks of contact tracers. *Los Angeles Times*

[B57] Powell M. Eligon J. (2020). Virus brings states to a standstill: sessions halt, budgets crater, plans wait; state legislatures are facing chaos amid the coronavirus crisis, with revenue projections suddenly expected to dip and major agendas put on hold. *New York Times.*

[B58] Richtel M. (2020). The pandemic may mean the end of the open-floor office as businesses contemplate the return of workers to their desks, many are considering large and small changes to the modern workplace culture and trappings. *New York Times.*

[B59] Roberts R. (2020). He’s in tune with the times. Randy Newman is reading, relaxing, even writing the odd ditty these days. *Los Angeles Times.*

[B60] Rodell B. (2020). How Australians seek connection in isolation, from virtual birthday parties to distanced neighborhood listening sessions, people are finding new ways to be together while alone. *New York Times.*

[B61] Saad N. (2020). Pandemic to hit ‘Grey’s Anatomy,’ amplifying COVID-19 stories from medical workers. *Los Angeles Times.*

[B62] Saldaña J. (2014). “Coding and analysis strategies,” in *The Oxford Handbook of Qualitative Research*, ed. Leavy P. (New York, NY: Oxford University Press).

[B63] Saldaña J. (2015). *The Coding Manual for Qualitative Researchers.* Thousand Oaks, CA: Sage.

[B64] Sawyer R. K. John-Steiner V. Csikszentmihalyi M. Moran S. Feldman D. H. Gardner H. (2003). *Creativity and Development.* New York, NY: Oxford University Press.

[B65] Smith P. (2020). Closed houses of worship served during 1918 flu pandemic. *Pittsburgh Post-Gazette.*

[B66] Sternberg R. J. Kaufman J. C. Roberts A. M. (2019). “16 The relation of creativity to intelligence and wisdom,” in *The Cambridge Handbook of Creativity*, eds Kaufman J. Sternberg R. (Cambridge: Cambridge University Press), 337–352. 10.1017/9781316979839.018

[B67] Sternberg R. J. Lubart T. I. (1999). “The concept of creativity: prospects and paradigms,” in *Handbook of creativity*, Vol. 1 ed. Sternberg R. J. (Cambridge: Cambridge University Press), 3–15. 10.1017/cbo9780511807916.003

[B68] Swed M. (2020). How will the L.A. Phil carry on amid COVID-19? Dudamel and Smith lay out a plan. *Los Angeles Times.*

[B69] Tanggaard L. (2013). The sociomateriality of creativity in everyday life. *Cult. Psychol.* 19 20–32. 10.1177/1354067x12464987

[B70] Tschorn A. (2020). ‘I haven’t had a job since March 13’: how costume designers are coping with COVID-19. *Los Angeles Times.*

[B71] Vygotsky L. S. (1971). *The Psychology of Art.* Cambridge, MA: The MIT Press.

[B72] Wantanabe T. (2020). USC’s first-ever online graduation: surreal pomp, unprecedented circumstances. *Los Angeles Times.*

[B73] Wheeler I. (2020). Shakespeare survived quarantine with a little help. From his patrons; our support system for creative professionals is abysmal. Artists shouldn’t feel bad about feeling uninspired during a crisis. *New York Times.*

[B74] Whip G. (2020). Yes, it’ll be different; the Emmy Awards are moving forward, even if the show has to be held virtually. What’s certain is it will have to change. *Los Angeles Times.*

[B75] Wilson M. (2020). The virus has wrecked some families. It has brought others closer. With little else to do, many people are savoring the time they have for simple pleasures like eating and dancing with the ones they love. *New York Times.*

[B76] Yang M. (2020). Resilience and meaning-making amid the COVID-19 epidemic in China. *J. Humanist. Psychol.* 60 662–671. 10.1177/0022167820929215

[B77] Yarrow A. (2006). Clifford Geertz, cultural anthropologist, is dead at 80. *New York Times.*

[B78] Yin R. K. (2018). *Case Study Research and Applications: Design and Methods.* Thousand Oaks, CA: SAGE.

